# CabriTrack: Accelerometer data for automated behavioural monitoring of grazing Creole goats

**DOI:** 10.1016/j.dib.2025.111431

**Published:** 2025-03-01

**Authors:** Laura Faillot, Willy Troupe, Mathieu Bonneau

**Affiliations:** a ASSET, UR0143, INRAE, 97170 Petit-Bourg, Guadeloupe, France; b PTEA, INRAE, Tropical Livestock Experimental Facility, 97170 Petit-Bourg, Guadeloupe, France

**Keywords:** Accelerometer, Behaviour, Annotation, Goats

## Abstract

The availability of sensors and AI-based methods offers new perspectives for monitoring animal behaviour. In particular, accelerometers can record individual acceleration data for weeks, which can then be used to identify the activity of the animal. Several research articles have demonstrated the capacity of this technology, particularly using machine learning or deep learning, for behaviour estimation. These techniques need high-quality datasets to train and validate the models, particularly with a great diversity of examples for each considered behaviour and recorded animals. The diversity of the data is an important prerequisite for deploying these solutions at a large scale. In this context, the dataset presented here contains more than 144 hours of tri-axial accelerometer data, collected from 59 different animals. The data were collected from March 2023 until March 2024. Two to five adult Creole goats were equipped with an accelerometer on one horn and allowed to graze in a small experimental pasture. While grazing, the behaviour of the animals was recorded with a CCTV camera. The videos were then manually annotated using the software Boris to identify the behaviour of each animal when it was possible to do so. Five behaviours were considered: ruminating/chewing, grazing, resting, displacement, and other, which includes behaviours such as scratching or fighting with a congener. Finally, the behaviour sequences were associated with the corresponding acceleration sequences based on a time synchronization procedure, so that each acceleration sequence is associated with one behaviour. This dataset can be used to train and evaluate any prediction methods for behaviour prediction from acceleration data using tri-axial accelerometers mounted on the horn of grazing Creole goats.

Specifications Table

**Table:** 

Subject	Signal processing, Systematics, Ecology and Behaviour
Specific subject area	Automatic animal behaviour monitoring using accelerometer sensors.
Type of data	Table
Data collection	Tri-axial accelerometers were set up on the horns of the adult Creole goats, which were free to graze in an experimental field. Their activities were recorded using a CCTV camera throughout the entire experiment, and the videos were manually annotated using the software Boris. We used a procedure to synchronize the time of the accelerometers with the video time to label the acceleration data. The result is a collection of acceleration sequences, each corresponding to a particular behaviour.
Data source location	*Institution :* PTEA, INRAE, 2024. Tropical Livestock Experimental Facility, doi.org/10.17180/50N1-KN86 *City/Town/Region :* Le Moule *Country:* Guadeloupe, French West Indies. *Latitude/Longitude:* 16.301894194741912, -61.32476183730542
Data accessibility	Repository name: data.gouv.fr Data identification number: https://doi.org/10.57745/CYF9QW Direct URL to data: https://entrepot.recherche.data.gouv.fr/dataset.xhtml?persistentId=doi:10.57745/CYF9QW Instructions for accessing these data: none
Related research article	*None*

## 1 Value of the Data

• The dataset is particularly valuable for its diversity, in total 59 animals are included, and its size, with more than 144 hours of annotated acceleration sequences.
• The dataset can be used to train and validate any methods for the prediction of animal behaviour from accelerometer.
• The trained method can then be used for monitoring animal behaviour and study the relationship between behaviour, health and welfare.
• AI based methods require large and diverse dataset to be used on a large scale, this dataset will contribute to the creation of such dataset.

## 2 Background

The importance of animal behaviour has increased in recent years, as it is a key variable in the management of animal health and welfare. Behaviour can be used to quantify and monitor animal welfare, an increasingly important economic issue, with consumers calling for a reconsideration of animals’ place in the livestock system [1]. Behaviour can also serve as a significant indicator of animal health, for example, by monitoring behavioural changes that may indicate health issues. This can be particularly useful for implementing rapid responses to health problems or targeted selective treatments, in either case reducing the use of chemical treatments. Furthermore, behaviour could be leveraged to develop new management options, such as selecting animals with specific behavioural traits. To be able to rely on behavioural information during the animals’ life, automatic monitoring methods have to be developed. Such method should allow transforming behaviour into quantitative variables, such as the time budget of the animals into different postures or activities. Combined with models, this information could be used to better characterize and improve the management of the animal's health and welfare. Accelerometers are an interesting tool for monitoring behaviour, particularly because it allows recording continuous individual acceleration data, that can be turned into behavioural insights. To be able to do that, training dataset have to be created, in order to define supervised inference methods.

For small ruminants, gastro-intestinal nematodes (GIN) are a major threat [2]. This situation becomes particularly difficult with the emergence of widespread anthelmintic resistance, possibly leaving breeders without solutions [3]. If we can detect symptoms of infestation through changes in behaviour, it could facilitate the practical application of targeted selective treatment methods, limiting treatment to infested animals only, rather than the entire flock. Conversely, it would be interesting to explore whether there are behavioural characteristics that increase the risk of larval ingestion, potentially allowing for the breeding of animals with a lower risk of infestation.

## 3 Data Description

**Fig. 1:**
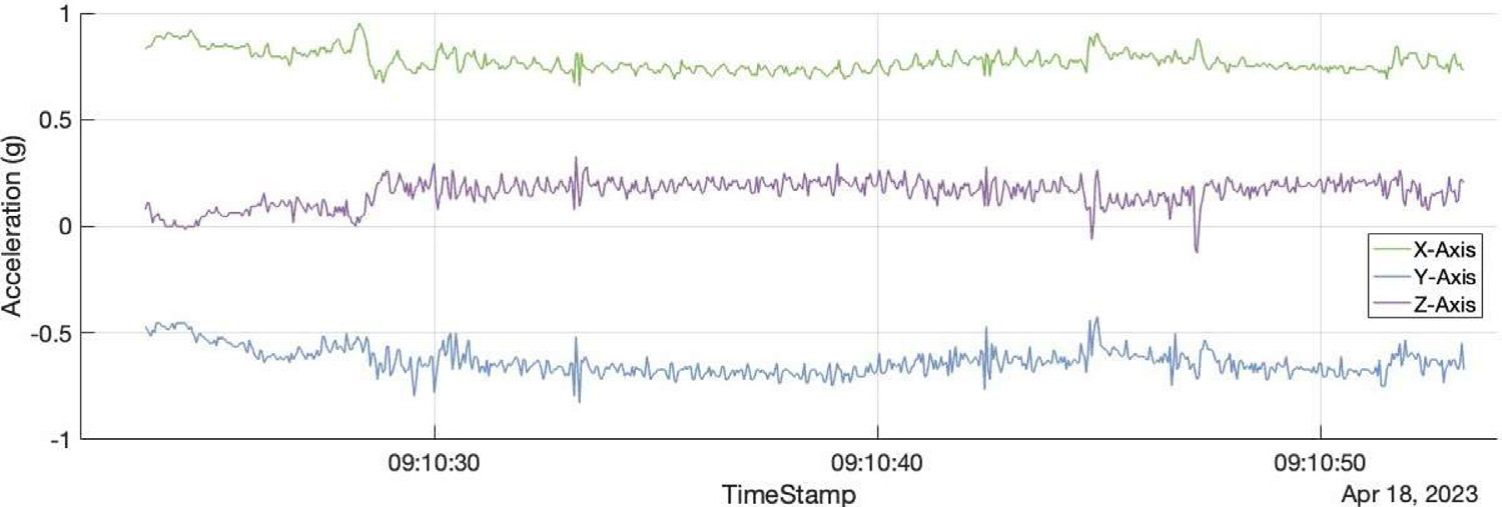
Example of the accelerations in the X, Y and Z axis for one sequence. The sequence is for animal 35, was recorded from April 18^th^ 2024, from 9:10:23 until 9:10:53 o'clock. The corresponding behaviour was Ruminating/Chewing. The acceleration is provided in units of gravity.

The dataset is organized as a single table, saved as a ‘txt’ file. Each row of the table provides the acceleration values over the three spatial dimensions (X, Y, Z), along with all the corresponding metadata: timestamp, animal ID, behaviour name, behaviour number, and sequence number. In total, the table contains 12,756,669 rows and 8 columns. The data are organized into sequences, i.e., a set of successive acceleration values from the same animal, performing one behaviour. For example, an animal grazing for 1 minute and then resting for 2 minutes creates two sequences. Given the sampling rate of the accelerometers was 25Hz, the first sequence represents 25*60 rows in the table, labeled with the grazing behaviour. The second sequence creates 25*60*2 rows in the table, associated with the resting behaviour. Each sequence is labeled with a unique sequence ID, provided in the column Sequence_num. In total, 16,500 sequences were collected. The column Time provides the timestamp of the sequence's acceleration values in the format ‘year-month-day hours:minutes:seconds’, or more precisely ‘yyyy-MM-dd hh:mm:ss’. The column Animal_id provides the animal number from which the sequence was recorded. Note that we did not provide the national identification number, but simply a fake ID, from 1 to 59, to allow for the selection of sequences from a specific animal. The columns Behaviour and Behaviour_num indicate the behaviour of the animal during the sequence. Behaviour uses a string format, while Behaviour_num uses an integer format. Behaviour 1 is Displacement, 2 is Grazing, 3 is Ruminating/Chewing, 4 is Other, and 5 is Resting. Finally, the other 3 columns, X, Y, and Z, are for the acceleration values along the three spatial axes (see description below). An example of a sequence is provided in Fig. 1.

## 4 Experimental Design, Materials and Methods

### 4.1 Accelerometers

**Fig. 2:**
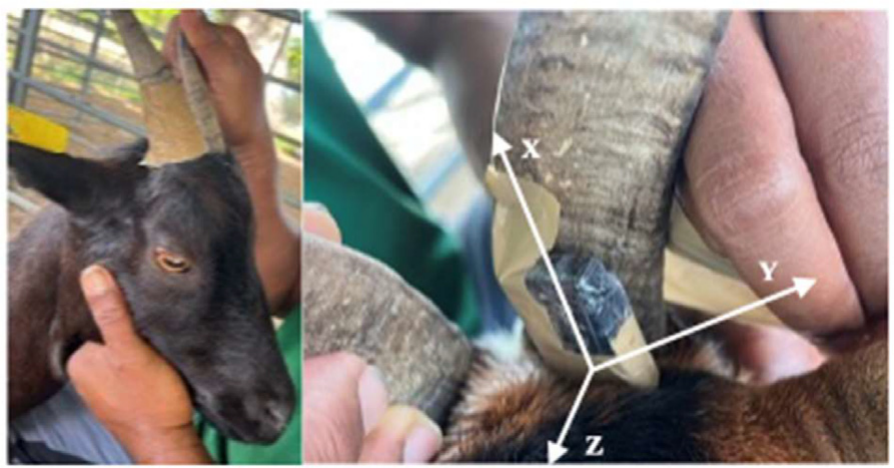
Location of the accelerometer on the animal's horn and of the three spatial axes.

Accelerometers from Axivity, model AX3, were used during the study. The accelerometers were set up with tape on the inside of the left horn, as viewed from an observer located in front of the animal (see Fig. 2). The X axis is along the horn, the Y axis along the back of the animal, and the Z axis is perpendicular to and on the same plane as the Y axis. Accelerometers recorded the acceleration at a frequency of 25Hz. We chose to use the horn instead of an ear tag or collar for simplicity. At the beginning of the project, basic identification collars were used, but it was not possible to fit the collar perfectly to the animals’ necks. As a result, the accelerometer often swung out of place. Additionally, the weight of the accelerometer could damage the animals' ears, potentially causing monitoring bias. In contrast, the accelerometer could be easily attached to the horn, where it remained securely in the same location for several days.

### 4.2 Experimental field and animals

**Fig. 3:**
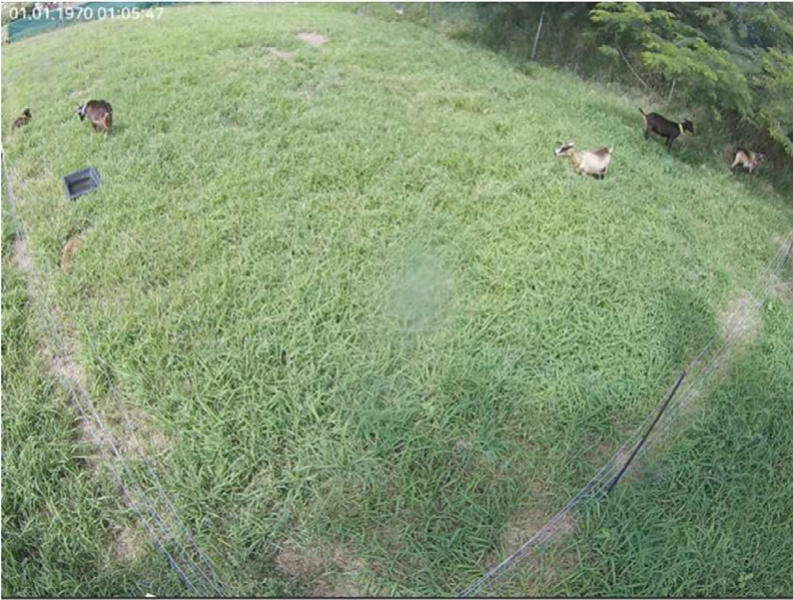
Illustration of the experimental field. The image was recorded with the CCTV camera used during the experiment.

A small experimental paddock of 20m x 10m was delimited with electric fences inside the farm. Two to five animals were equipped with accelerometers and introduced into the field for an observation period of 6 to 8 hours. The experiment started in March 2023 and ended in March 2024. During this period, only 29 days of observations were performed, depending on the availability, of forage and technicians. Although it was not the original plot of the animals, it provided similar grazing conditions to what the animals were exposed to all year round. Animals were healthy and exhibited normal behaviour during the observation days. Adult lactating and pregnant Creole goats were used for the experiment; these animals were raised outdoors all year round. A water bucket was placed inside the pasture, (Fig. 3).

### 4.3 Video monitoring

Animals were monitored with a CCTV camera connected to a Raspberry Pi that saved the footage on a hard drive. Videos were collected at the end of each day of observation. The Raspberry Pi was set up to record 30-minute segments. Video resolution was 2592 × 1944 pixels and recorded in h264 format, which was then converted to mp4 to be readable on any computer. Each animal wore a collar with different colors to make identification easier when analyzing the footage. An experimental sheet was used each time an animal was equipped with an accelerometer to record the animal's color, the color of its collar, and the accelerometer used.

### 4.4 Time synchronization

It is important to synchronize the timestamps of the accelerometers and the camera. The accelerometers were automatically synchronized to UTC time using a computer with the Open Movement software provided by the accelerometer's manufacturer. The CCTV camera was not connected to the internet, and the camera time was synchronized after recording. We simply presented the clock of a smartphone close to the camera while it was recording. An image was then selected where the clock was visible. The camera timestamp is displayed on all the images in the top left corner (see Annex 1). We then computed the offset between the camera and UTC time using the selected image.

For each recorded 30-minute video, the camera time at the start and end of the video was manually read from the footage, saved, and converted to UTC time. Taking into account the frame rate of the camera, each video frame was then associated with a UTC time, which could be compared to the accelerometer time.

### 4.5 Time drift

Unfortunately, the clocks of electronic devices are not always accurate, and one hour of data recording could correspond to one hour plus or minus a few seconds for the two sensors (camera or accelerometer). To compute the time drift, it is important to determine the duration of the experiment from a reliable source and then compare it to the duration obtained from the sensors. In our case, the reliable source of time was a smartphone connected to the internet.

To compute the time drift, we created reference points for each sensor. For the camera, we used the same method as for time synchronization. The clock of a smartphone was displayed to the camera at the beginning and end of the experiment to compute the true duration. As explained before, two images where the smartphone's clock was visible were selected, one at the beginning and one at the end of the experiment. Then, the camera's experimental duration was also computed using the camera timestamp on these two images. We simply computed the difference between the two durations and adjusted the frame's UTC timestamp computed earlier accordingly. For example, if the difference between the two durations was equal to 1s, i.e. the time drift was equal to d = 1s. Then, each frame's UTC timestamp was changed to $New_{UTC}=\;Old_{UTC}+d\;/\;n_{Frame},$ where $New_{UTC}$ is the frame's UTC timestamp, corrected with the time drift. $Old_{UTC}$ is the frame's UTC timestamp computed during the time synchronization procedure, and $n_{Frame}$is the number of frames recorded during the duration of the experiment. An example of the time drift computation for the camera is available in Annex 1.

For the accelerometers, we created a distinctive acceleration pattern that started at a known UTC time. The pattern consisted of shaking the accelerometers vigorously for 5 seconds and then resting for 10 seconds, repeated 5 times. The UTC time at the beginning of the pattern was recorded with a smartphone to compute the true duration. This pattern was implemented at the beginning and end of the experimentation. Similarly to the camera, the true experimental duration was obtained from the recorded UTC time and compared to the duration computed from the accelerometer timestamps. The accelerometer timestamps were then adjusted accordingly to account for the time drift. An example of the time drift computation is available in Annex 2.

### 4.6 Manual labelling of behaviours

At this stage, each video frame and acceleration data point are synchronized with UTC time. The final step involves integrating behavioral information into the acceleration data. We used the Boris software for video labeling. As previously mentioned, each animal was fitted with a collar of a distinct color, which facilitated the labeling process. One trained observer labeled all the videos. However, there were instances where parts of the videos remained unlabeled due to difficulties in detecting the animal's identity or the nature of the behavior. For example, when the animals were too far from the camera or in the shade, it was challenging to accurately identify the collar colors or observe the behavior. Additionally, there were cases where the animals' heads and/or collars were not clearly visible, preventing accurate behavioral estimation. Some videos were also excluded from the dataset, for example when animals where not present or a too large proportion of the video was too challenging to label, e.g. all animals were simply at the opposite side of the camera. In total, 60% of the videos where not considered for labelling.

When conditions allowed for reliable identification of the animal and its behavior, the observer categorized the behavior into one of five types: Grazing, Displacement, Ruminating/Chewing, Resting, and Other. A detailed description of these behaviors is provided in Table 1.

**Table 1:** Detailed ethogram used for the video labelling.

Comportements	Descriptions
**Grazing**	The animal is grazing with the head down. Between grazing sequences, the animal can pull its head up for chewing. The chewing sequences that last less than 5 seconds were labelled as Grazing, and as Ruminating/Chewing for longer sequences
**Displacement**	The animal is moving, either walking or running inside the pasture but not grazing. It can be for example to move to the water bucket. Transitions between standing/lying and lying/standing were also labeled Displacement.
**Ruminating/Chewing**	The animal is either standing or lying, with the head straight and ruminating or chewing, with a moving jaw. During two Ruminating/Chewing sequences, the animal can stop the jaw movement. When the sequence was shorter than 3 seconds, it was labeled as Ruminating/Chewing. Longer sequences were labeled as Resting.
**Resting**	The animal is either standing or lying, with the head straight, or set down on the ground, with no activity. Sequences where the animal was on alert, with quick changes of the head direction was not labeled as resting, but Other. Same when the animal was bleating.
***Other***	All other observed behaviours. It includes, bleating, alarm, eating the hedges, scratching, social interactions, drinking, urinate or defecate.

**Fig. 4:**
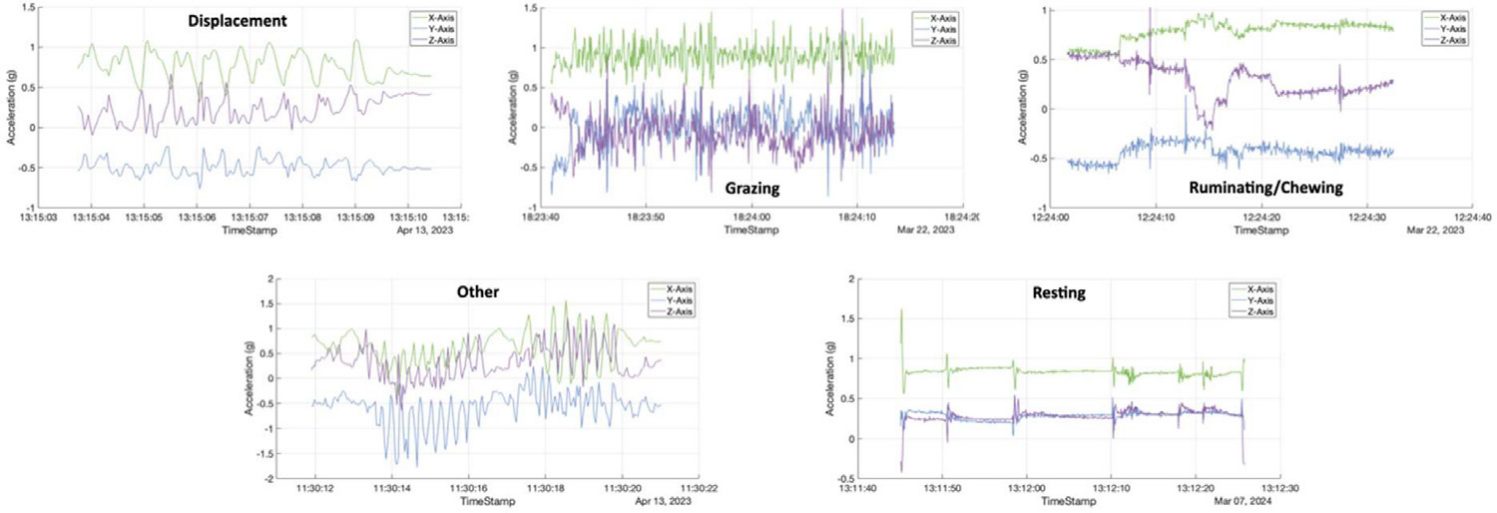
Examples of raw acceleration sequences.

Once the video labeling was completed, it was converted to a CSV file using Boris. The CSV file was organized by sequences, each corresponding to a single animal performing a specific behavior, along with the start and stop times of each sequence in the video. We then applied the previously described method to match the start and stop times of these sequences with the accelerometer data. Examples of acceleration sequences for the different behaviors are provided in Fig. 4. In total, 59 different goats were included into the dataset. Each goat contributed in average to 1.7% of the dataset (SD=0.99, Min=0.000776, Max=4.741959).

## Limitations

The first limitation is the heterogeneity of the dataset. Goats predominantly spent their time grazing or resting, resulting in a dataset that is heavily skewed towards these behaviours. The total durations of sequences are as follows: Displacement – 0.6 hours, Grazing – 67.7 hours, Ruminating/Chewing – 10.6 hours, Other – 6.2 hours, and Resting – 59.5 hours.

The second limitation concerns the time synchronization process. Despite careful attention, perfect synchronization between the camera and accelerometers is challenging to achieve. We assumed that time drift was consistent throughout a day of experiment; however, it could vary slightly due to factors such as temperature or humidity. This drift is difficult to measure and account for precisely in our procedure. Consequently, small time discrepancies between the synchronized camera and accelerometer data were observed. Although generally not problematic, these discrepancies may cause the start or end of sequences to include acceleration data from different behaviours. This may be particularly challenging for short behaviours, such *Other*.

The third limitation is on the accelerometer's location (horn here). Caution should be applied if the dataset is mixed with acceleration data coming from other locations, such as ear tag or collar. There is no guaranty that the acceleration signal would be comparable in the different activities.

To the best of our knowledge, these limitations are inherent to this type of procedure and complicate the development of automatic prediction methods.

## Ethics Statement

The experiment describe in this article were reviewed and authorized by the Fench Measurements and observation on animals were performed in accordance with the current law on animal experimentation and ethics (#69-2023-3_v2 from the Animal Care and Use Committee of French West Indies and Guyana) under the direction of N. MINACHY (INRAE – PTEA).

## CRediT authorship contribution statement

**Laura Faillot:** Writing – original draft, Data curation, Methodology. **Willy Troupe:** Conceptualization, Resources, Supervision. **Mathieu Bonneau:** Writing – review & editing, Supervision, Funding acquisition, Conceptualization.

## Data Availability

data.gouvCabriTrack: Accelerometer data for automated behavioural monitoring of grazing goats (Original data)

## Appendix Supplementary materials

Image, image 1

Image, image 2

Image, application 3

Image, application 4
