# Supplementary figures and images for: CabriTrack: Accelerometer data for automated behavioural monitoring of grazing Creole goats
Source: Data Brief. 2025 Mar 1;59:111431. doi: 10.1016/j.dib.2025.111431 (PMC11953975; doi:10.1016/j.dib.2025.111431)

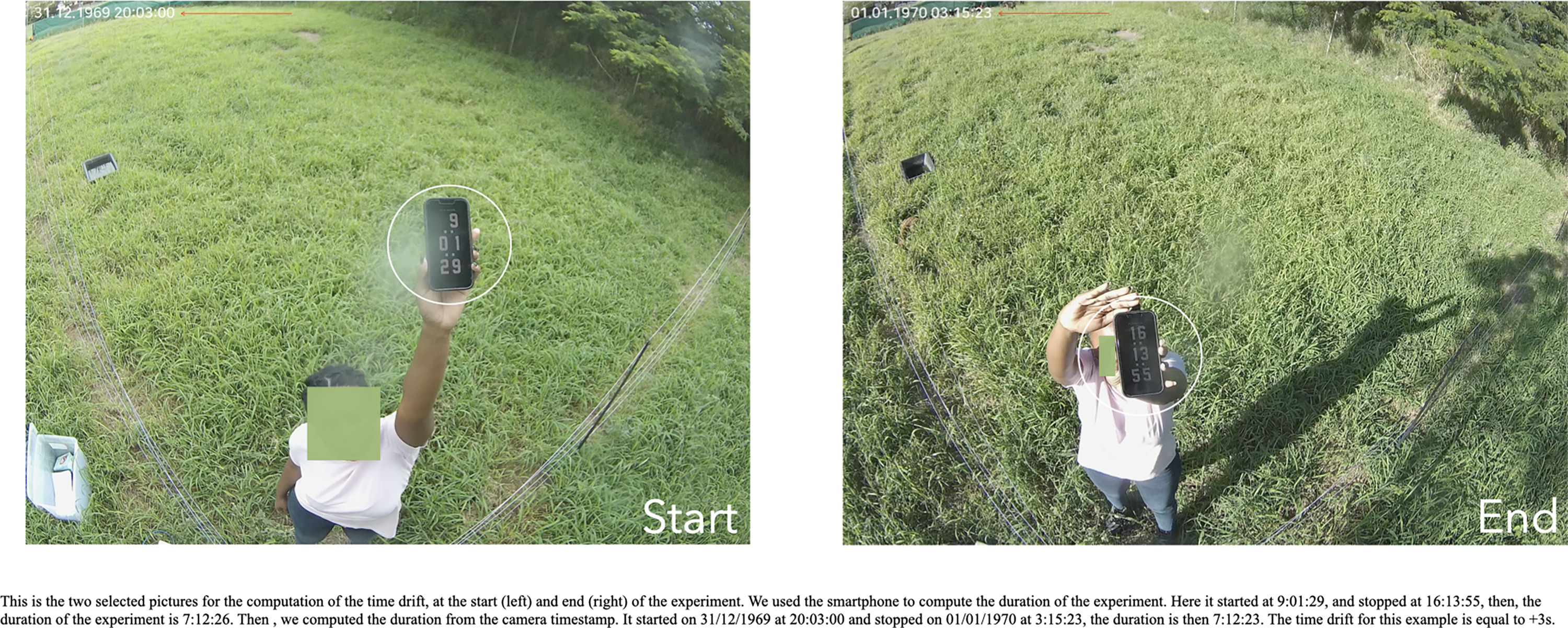

Supplement: Supplementary file 1 [file mmc1.jpg]

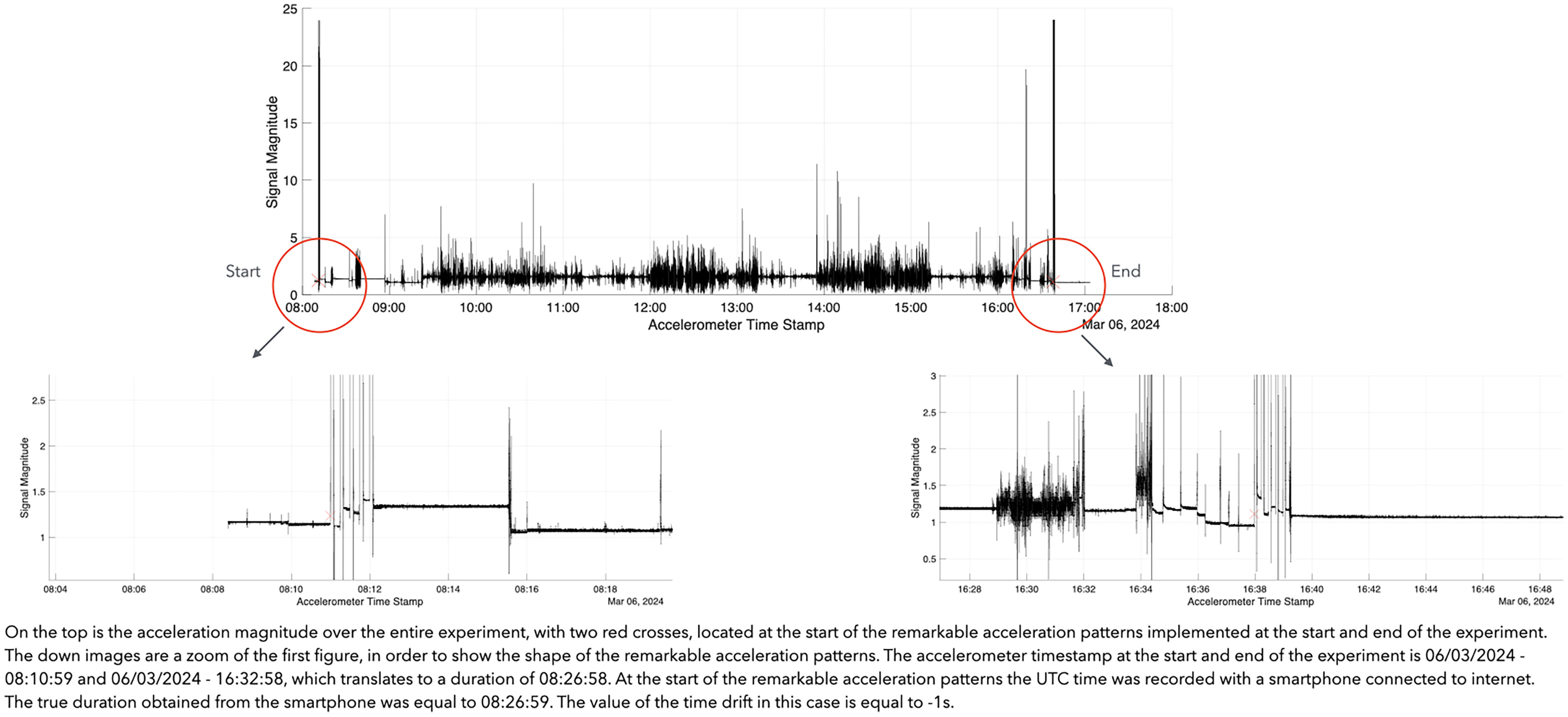

Supplement: Supplementary file 2 [file mmc2.jpg]
