# Supplementary material for: CabriTrack: Accelerometer data for automated behavioural monitoring of grazing Creole goats
Source: Data Brief. 2025 Mar 1;59:111431. doi: 10.1016/j.dib.2025.111431 (PMC11953975; doi:10.1016/j.dib.2025.111431)
Supplement: Supplementary file 3 [file mmc3.docx]

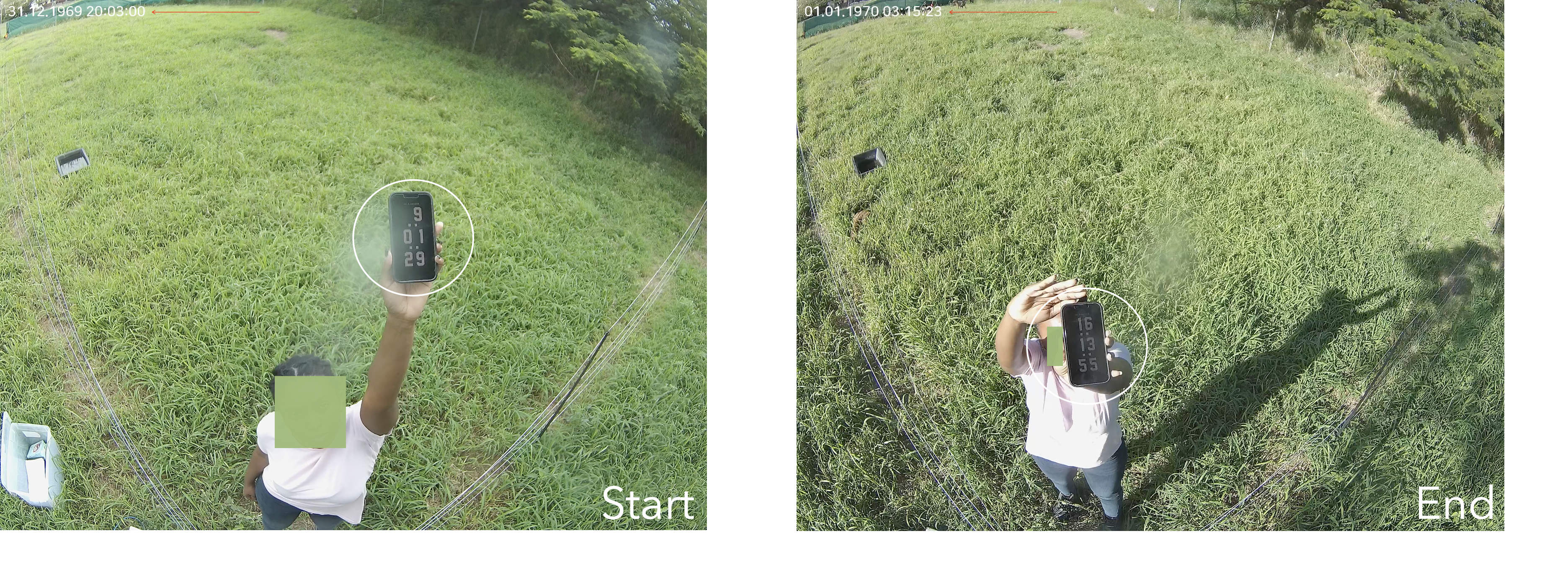


**Figure 1:** This is the two selected pictures for the computation of the time drift, at the start (left) and end (right) of the experiment. We used the smartphone to compute the duration of the experiment. Here it started at 9:01:29, and stopped at 16:13:55, the duration of the experiment is thus 7:12:26. Then, we computed the duration from the camera timestamp. It started on 31/12/1969 at 20:03:00 and stopped on 01/01/1970 at 3:15:23, the duration is thus 7:12:23. The time drift for this example is equal to +3s.
