# Supplementary material for: CabriTrack: Accelerometer data for automated behavioural monitoring of grazing Creole goats
Source: Data Brief. 2025 Mar 1;59:111431. doi: 10.1016/j.dib.2025.111431 (PMC11953975; doi:10.1016/j.dib.2025.111431)
Supplement: Supplementary file 4 [file mmc4.docx]

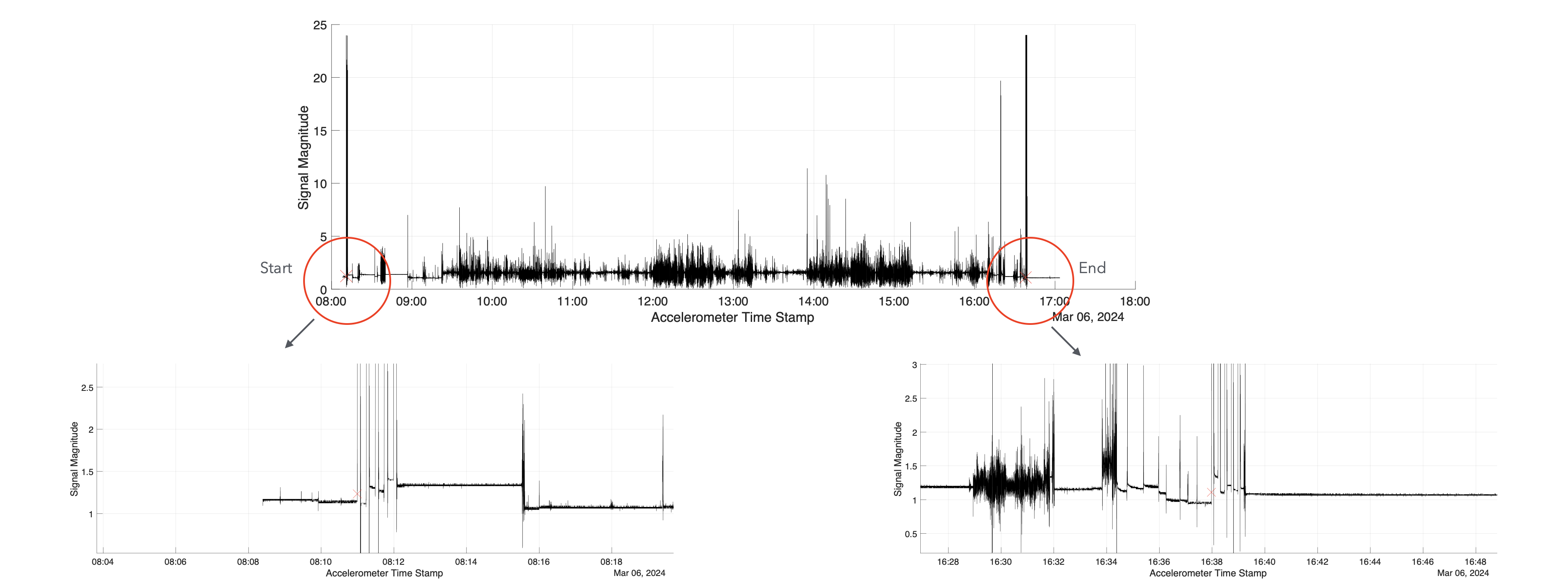


**Figure 1:** On the top is the acceleration magnitude over the entire experiment, with two red crosses, located at the start of the remarkable acceleration patterns implemented at the start and end of the experiment. The down images are a zoom of the first figure, in order to show the shape of the remarkable acceleration patterns. The accelerometer timestamp at the start and end of the experiment is 06/03/2024 - 08:10:59 and 06/03/2024 - 16:32:58, which translates to a duration of 08:26:58. At the start of the remarkable acceleration patterns the UTC time was recorded with a smartphone connected to internet. The true duration obtained from the smartphone was equal to 08:26:59. The value of the time drift in this case is equal to -1s.
